# Temperature-Compensated Vector Bending Sensor with Double-Cladding Fiber Assisted Mach–Zehnder Interferometer

**DOI:** 10.3390/biomimetics11020100

**Published:** 2026-02-02

**Authors:** Wenchao Li, Hongye Wang, Shuqin Wang, Xiangwei Hao, Yan Bai, Jian Xing, Xuelan He

**Affiliations:** 1 College of Computer and Control Engineering, Northeast Forestry University, Harbin 150040, China; liwenchao@nefu.edu.cn (W.L.); wangsq@nefu.edu.cn (S.W.); baiyan@nefu.edu.cn (Y.B.); xj@nefu.edu.cn (J.X.); 2 School of Electronic and Information Engineering, Ningbo University of Technology, Ningbo 315211, China; wanghongye92@nbut.edu.cn; 3 College of Civil Engineering and Transportation, Northeast Forestry University, Harbin 150040, China; hxw_75@nefu.edu.cn

**Keywords:** vector bending sensor, fiber-optic sensor, Mach-Zehnder interferometer

## Abstract

Vector bending sensing is an important research direction in the field of bionic robot design. A vector bending sensor with temperature compensation based on Mach–Zehnder interferometer (MZI) is proposed and experimentally investigated. The MZI is implemented using an off-axis splice between a single-mode fiber (SMF) and a double-cladding fiber (DCF). The proposed sensor is analyzed comprehensively from the perspective of theoretical analysis and experimentally demonstrated. It reaches a high curvature sensitivity as high as −8.311 nm/m^−1^ and a compact size as small as 3 mm, while keeping the capability of direction sensing and temperature compensation. The proposed vector bending sensor has a good potential for accurate curvature measurement due to its high accuracy, multifunction, low cost and, compact size.

## 1. Introduction

In recent years, the bending sensor has been highly demanded in the fields of bionic robot design [1,2]. Compared with traditional electrical sensors, the optical fiber sensor has the following advantages: small volume, anti-electromagnetic interference, corrosion resistance, and low maintenance cost [3,4,5]. Therefore, the fiber optic sensor is a diversified and excellent sensing technology, which can deal with a variety of application scenarios.

With the rapid development of optical fiber technology, a variety of optical fiber devices have been used in bending sensing applications [6,7,8]. Fiber bending sensors mainly include three categories: fiber Bragg grating (FBG), long period fiber grating (LPFG), and fiber modal interferometer [9,10,11,12]. Among them, the bending sensors based on FBG, which is most used in engineering, need special equipment and particular designs to obtain non-zero bending sensitivity [13,14]. The bending sensor of the optical fiber modal interferometer, including Mach–Zehnder interferometer (MZI) and Fabry–Perot interferometer (FPI), has the advantages of simple structure, low cost, and high sensitivity [15,16,17]. The bending sensitivity of the optical fiber bending sensor based on FPI cavity is usually limited by the small size of the FP cavity [15]. Bending sensors based on MZI have been widely used in recent years [17,18]. A variety of sensors based on MZI structures have been proposed, including single-mode fiber (SMF) and multimode fiber (MMF) SMF structures, SMF thin core fiber SMF structures, and SMF multicore fiber SMF structures [6,19,20]. In addition to the aforementioned structures, S-tapered fibers and non-adiabatic tapers have also been widely used in MZI-based bending sensors, which achieve mode coupling through structural tapering to realize sensing functions [21,22]. Although these structures have their own advantages, most of them are easily influenced by external parameters, especially temperature. More importantly, in some applications such as structural health monitoring, the direction of deformation is also very important. Therefore, how to identify the bending direction without the influence of ambient temperature is also an important research topic.

In this paper, a temperature-compensated vector bending sensor based on the double-cladding fiber (DCF) assisted Mach–Zehnder interferometer (DMZI) is proposed and demonstrated. With a suitably designed misalignment and length of DCF, a modal interferometer based on the core mode and excited cladding mode is established with good reliability. Compared with conventional MZI composed of SMFs dislocation fusion, the proposed sensor’s structure achieves better curvature sensing performance, as the special structure of DCF enables stable transmission of the cladding mode inside the inner cladding. The asymmetric structure of the sensor makes it sensitive to the bending direction, so vector sensing is realized. Moreover, the existence of different modes in the inner cladding enables the sensor to compensate for the change in ambient temperature. In addition, the sensor proposed in this paper has the advantages of its simple manufacturing process and low cost, so it is a cost-effective vector bending sensing scheme in the field of bionic robot design.

## 2. Materials and Methods

### 2.1. Structure of DMZI

**Figure 1:**
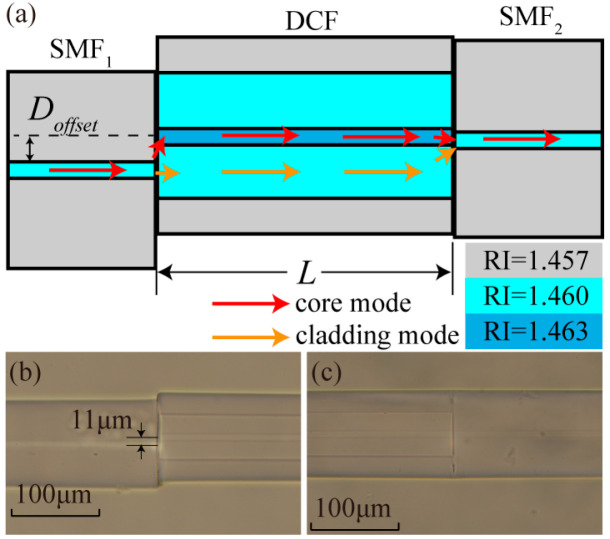
(**a**) Schematic diagram of DMZI sensor. Microscope image of the fusion of single-mode fiber and double clad fiber at the left (**b**) and right (**c**) ends of the sensor.

In Figure 1, the proposed DMZI sensor is composed of a segment of DCF and SMF connected on both sides of it. The core and cladding diameters of SMF are 8.3 μm and 125 μm, respectively, and the corresponding refractive indices are 1.454 and 1.447, respectively. The diameters of the core, inner cladding, and outer cladding of DCF are 8.3 μm, 62.5 μm, and 125 μm, respectively, and the refractive indexes (RIs) are 1.459, 1.452, and 1.447, respectively. One end of the DCF and SMF_1_ is misaligned fusion with a large core offset (*D*_offset_), and there is a very small displacement between the other end of DCF and the core of the SMF_2_, so that the light in the inner cladding of DCF can return to the core of SMF_2_, as shown in Figure 1.

### 2.2. Principle of DMZI

Figure 1 shows the schematic diagram of the DMZI configuration. The length of DCF is *L*. The core offset between DCF and SMF_1_ is *D*_offset_. As shown in Figure 1a, light transmits in the core of SMF_1_ as a core mode. Due to the offset of DCF and SMF_1_ cores, light enters the inner cladding of DCF which excites cladding modes. These excited cladding modes propagate in the inner cladding of DCF and enter the core of SMF_2_. As the effective RI and optical path of light propagating in core mode and cladding mode are different, a modal interferometer is formed. The interfering intensity of the modal interferometer can be given by [23]

(1) $$I=I_{\mathrm{core}}+\sum_{i=1}^{N}I_{\mathrm{inner}-\mathrm{cladding}}^{i}+2\sum_{i=1}^{N}\sqrt{I_{\mathrm{core}}I_{\mathrm{inner}-\mathrm{cladding}}^{i}}\cos\left(\Delta \varphi_{i}\right)$$

where *I*_core_ represents the intensity of the core mode, *I^i^*_inner-cladding_ represents the intensity of the *i*th cladding mode, Δ*φ_i_* is the phase difference between the core mode and the *i*th cladding mode, and *N* is the total number of the excited cladding mode. Δ*φ_i_* can be expressed as follows:

(2) $$\Delta \varphi_{i}=\frac{2\pi \left(n_{\mathrm{core}}-n_{\mathrm{inner}-\mathrm{cladding}}^{i}\right)L}{\lambda }$$

where *n*_core_ and *n^i^*_inner-cladding_ represent the effective RI of the core mode and the *i*th cladding mode, respectively. *λ* is the free space wavelength. According to Equation (2), an interference dip will appear when the phase difference Δ*φ_i_* meets the condition of Δ*φ_i_* = (2m + 1)π. The *m*th interference dip can be given by

(3) $$\lambda_{\mathrm{dip},m}=\frac{2\left(n_{\mathrm{core}}-n_{\mathrm{inner}-\mathrm{cladding}}^{i}\right)L}{2m+1}$$

**Figure 2:**
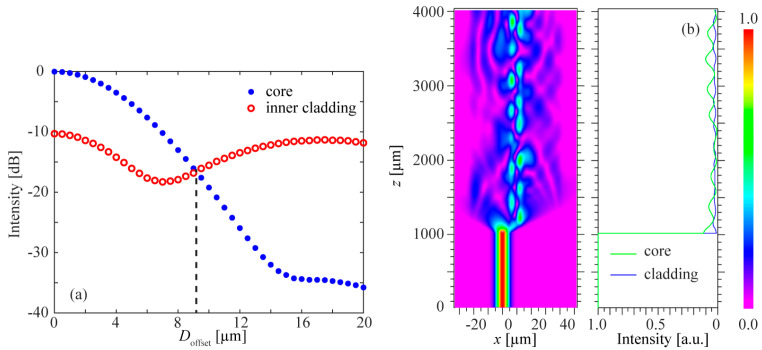
(**a**) Light intensity in core and inner cladding under different core offset (Doffset). (**b**) The simulation results of optical field intensity distribution of DMZI when the Doffset is 9.2 μm.

To study the influence of core offset of DCF and SMF on modal interferometer, beam propagation method (BPM) is used to carry out numerical simulation. The optical fiber parameters of SMF and DCF are described in Section 1, and the simulation length of DCF is 3 mm. When the core offset (*D*_offset_) is set as 0 μm ~ 20 μm, the simulation results are shown in Figure 2. From Figure 2a, it can be observed that when *D*_offset_ is 9.2 μm, the energy in the core and inner cladding is approximately equal. At this time, the effect of the mode interferometer is the best. In the BPM simulation, the cladding light intensity refers to the sum of the light intensities of all excited cladding modes in the DCF inner cladding region (calculated using the regional light field intensity integration method), and the core light intensity corresponds to the result of the light intensity integration in the DCF core region. The sum of the two is lower than the incident light intensity, mainly because misalignment welding causes part of the light to leak into the outer cladding/air and due to the intrinsic absorption loss of the material. This law is consistent with the trend of experimental transmission loss. When *D*_offset_ is 9.2 μm, the proportions of light intensity in the core and inner cladding are approximately equal. The energy balance makes the interference contrast reach the maximum value, which is the optimal interference condition. It should be noted that when *D*_offset_ = 9.2 μm, the condition “core mode optical energy ≈ inner cladding mode optical energy” is satisfied in BPM simulation. However, during the actual fusion splicing process, affected by process factors such as the flatness of the fiber end face and the precision of the fusion splicing equipment, through multiple experimental verifications, we determined that 11 μm is the actual optimal experimental parameter.

The simulation results of optical field intensity distribution of DMZI when the *D*_offset_ is 9.2 μm is shown in Figure 2b. As the misaligned distance between the two fiber cores increases, the transmission spectrum loss increases obviously as the offset increases, because more light enters the inner cladding, and less energy is coupled into the core of the DCF. However, with the increase in energy in the inner cladding, more modes are excited in the inner cladding to form the modal interferometer.

The free spectral range (FSR) of the proposed sensor can be calculated as follows:

(4) $$\Delta \lambda_{m}=\frac{\lambda_{m}\lambda_{m-1}}{\left(n_{\mathrm{core}}-n_{\mathrm{inner}-\mathrm{cladding}}^{i}\right)L}$$

**Figure 3:**
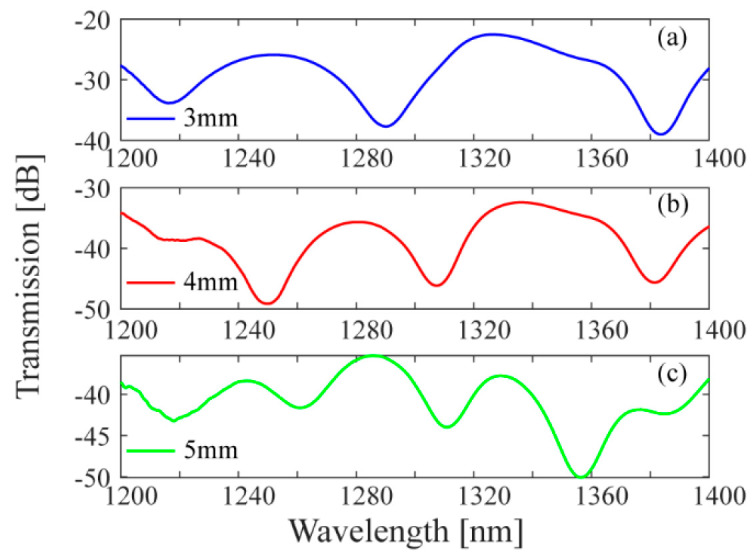
Spectral test results with sensor lengths of 3 mm (**a**), 4 mm (**b**), and 5 mm (**c**), respectively.

Based on the above theoretical analysis, the transmission spectrum (1200–1400 nm) of sensors composed of DCF with different lengths are experimentally studied. The lengths of DCF are 3 mm, 4 mm, and 5 mm, respectively. The measurement results are shown in Figure 3. It can be observed that, with the increase in DCF length, the intensity of transmission spectrum decreases, and the FSR of the proposed modal interferometer decreases gradually.

**Figure 4:**
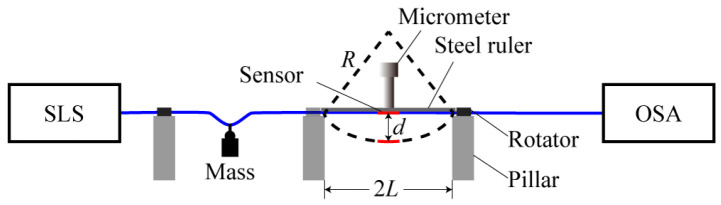
Schematic diagram of bending sensing experimental setup.

After the sensor is bent at different bending directions, its effective RI will change as follows due to the elastic optical effect [24],

(5) $$n\left(C,\theta \right)=n\left(0,0\right)\left[1+\frac{C}{\rho }D_{\mathrm{offset}}\sin\left(\theta \right)\right]$$

where *ρ* is the elastic optic coefficient, *θ* is the angle of bending direction, *n*(*C*, *θ*) and *n*(0,0) are the effective RI at initial position and at curvature *C* and rotation angles *θ*, respectively. It should be noticed that bending in different directions is realized and controlled by a rotator in the experimental setup as described in Figure 4. Therefore, the angle of bending direction angle *θ* is equal to the rotation angles of the rotator. By substituting Equation (5) into Equation (3), the variation in the wavelength of the interference dips in the transmission spectrum of the sensor with curvature *C* and rotation angle *θ* can be obtained:

(6) $$\lambda_{dip,m}\left(C,\theta \right)=\frac{2L}{2m+1}\left[n_{\mathrm{core}}\left(0,0\right)-n_{\mathrm{inner}-\mathrm{cladding}}^{i}\left(0,0\right)\right]\left[1+\frac{C}{\rho }D_{\mathrm{offset}}\sin\left(\theta \right)\right]$$

The vector bending sensitivity *S_C_* of the interference dip in response to rotator angles and curvature variation can be expressed by

(7) $$S_{C}=\frac{d\lambda_{\mathrm{dip},m}\left(C,\theta \right)}{dC}=\frac{2D_{\mathrm{offset}}L}{\left(2m+1\right)\rho }\left[n_{\mathrm{core}}\left(0,0\right)-n_{\mathrm{inner}-\mathrm{cladding}}^{i}\left(0,0\right)\right]\sin\left(\theta \right)$$

It can be inferred from Equation (7) that the bending sensitivity of the sensor presents sinusoidal variation with the change in rotation angle *θ*.

## 3. Results

The schematic diagram of vector bending sensing experimental setup is shown in Figure 4. It consists of a super-continuum light source (SLS, SC-5, YSL Photonics, Inc., Wuhan, China), an optical spectrum analyzer (OSA, AQ6317b, Agilent Tech. Inc., Beijing, China), and two settings of 360° rotators fixed on two pillars.

**Figure 5:**
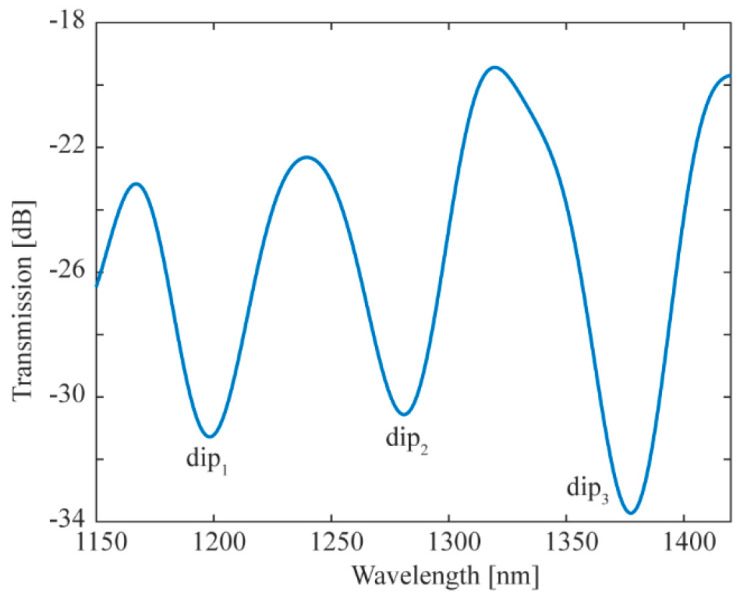
The transmission spectrum of sensor with sensor length of 3 mm and core offset of 11 μm.

A vector bending sensor with DMZI is fabricated as shown in Figure 1. The length of DCF is 3 mm, and the core offset is 11 μm. The transmission spectrum of the sensor is shown in Figure 5. The sensor is placed at the center of the two pillars. The two ends of the sensor are, respectively, connected with the SLS and the OSA through SMF. In the experiment, the steel ruler is deformed by the screw micrometer, which makes the sensor bend. The relationship between the curvature (*C*) applied on the sensor and the displacement (*d*) of the screw micrometer is as follows:

(8) $$C=\frac{1}{R}=\frac{2d}{d^{2}+L^{2}}$$

where *L* = 6.8 cm is half the spacing between the two pillars, *R* represents the bending radius of the sensor. In this experiment, the displacement (*d*) changes from 0 mm to 3.0 mm with a step of 0.2 mm. According to Equation (8), the curvature *C* varies from 0 m^−1^ to 1.3 m^−1^.

**Figure 6:**
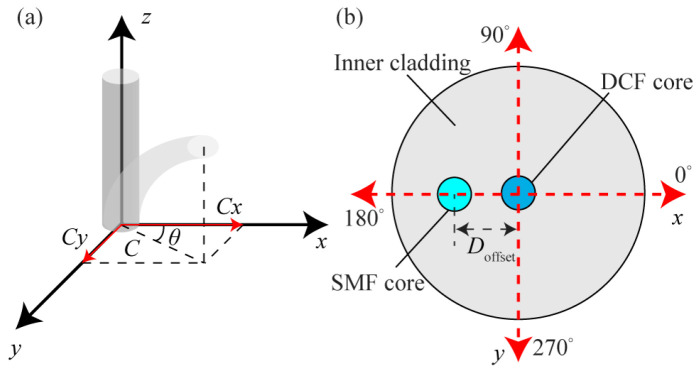
Schematic diagram of the bending direction defined in the coordinate system (**a**), and the cross section of DCF with the rotation angle (**b**).

The schematic diagram of the bending direction angle of the sensor is shown in Figure 6. Figure 6a is the bending direction defined in the coordinate system and Figure 6b is the cross section of the DCF with the rotation angle. When a certain curvature *C* is applied to the sensor at the rotation angle *θ*, the curvature in this direction can be decomposed into the x-axis and y-axis directions [25]. *C_x_* and *C_y_* are used to represent the bending components of the sensor in the x-axis and y-axis, respectively.

In practical terms, due to the thermo-optic effect, the interference dip will always be interfered with by the temperature when measuring curvature. Therefore, the cross sensitivity of temperature and curvature is the key to determine the accuracy of the sensor. As mentioned above, the inner cladding of double-cladding contains many modes, and different modes have different sensitivities to bending and temperature—specifically, low-order cladding modes propagate close to the DCF core, exhibiting high bending sensitivity but weak temperature response, while high-order cladding modes propagate away from the core, showing low bending sensitivity but prominent temperature sensitivity due to the enhanced thermo-optic effect. The transmission spectrum of the modal interferometer composed in the sensor is formed by the superposition of multiple interference dips. Using the unique properties of the sensor, the vector bending and temperature change can be measured at the same time through the wavelength change in different dips. The shifts in the dips can be written in matrix as follows:

(9) $$\left(\begin{array}{c} \Delta \lambda_{1}\\ \Delta \lambda_{2}\\ \Delta \lambda_{3} \end{array}\right)=\left(\begin{array}{ccc} k_{1x} & k_{1y} & k_{1T}\\ k_{2x} & k_{2y} & k_{2T}\\ k_{3x} & k_{3y} & k_{3T} \end{array}\right)\left(\begin{array}{c} C_{x}\\ C_{y}\\ \Delta T \end{array}\right)=K\left(\begin{array}{c} C_{x}\\ C_{y}\\ \Delta T \end{array}\right)$$

where *k_ix_* and *k_iy_* (*i* = 1,2,3) are the x-axis and y-axis curvature sensitivity coefficients for dip*_i_*, respectively. *k_iT_* is the temperature sensitivity coefficient for dip*_i_*. *K* is the coefficient matrix composed of *k_ix_*, *k_iy_*, and *k_iT_*. Then, the x and y components of curvature and temperature can be calculated by

(10) $$\left(\begin{array}{c} C_{x}\\ C_{y}\\ \Delta T \end{array}\right)=K^{-1}\left(\begin{array}{c} \Delta \lambda_{1}\\ \Delta \lambda_{2}\\ \Delta \lambda_{3} \end{array}\right)$$

Then, the vector curvature can be given by the curvature and the rotator angle:

(11) $$\left\{\begin{array}{c} C^{2}=C_{x}^{2}+C_{y}^{2}\\ \theta =\tan^{-1}\frac{C_{y}}{C_{x}} \end{array}\right.$$

**Figure 7:**
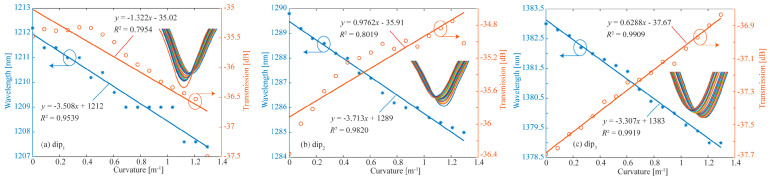
At T = 20 °C, the relationship between curvature and spectral change in dip1 (**a**), dip2 (**b**), and dip3 (**c**) in x-direction.

To investigate the temperature compensation and vector bending characteristics of the proposed DMZI sensors, the following experiments are demonstrated. Firstly, the curvature changes in the dip*_i_* in the x-axis and y-axis at room temperature are measured, respectively. Secondly, the changes in the dip*_i_* with temperature are measured. Then, the coefficient matrix *K* is obtained. At *T* = 20 °C, the relationship between curvature and spectral change in dip_1_ (a), dip_2_ (b), and dip_3_ (c) in the x-direction is shown in Figure 7. The curvature sensitivities of three interference dips are calculated as *k*_1_*_x_* = −8.311 nm/m^−1^, *k*_2_*_x_* = −6.459 nm/m^−1^ and *k*_3_*_x_* = −5.569 nm/m^−1^ in the x-direction, respectively. With the increase in curvature, the three dips shift to shorter wavelengths. The spectral loss of the dip_1_ increases, and the dip_2_ and dip_3_ decrease with the increase in curvature.

**Figure 8:**
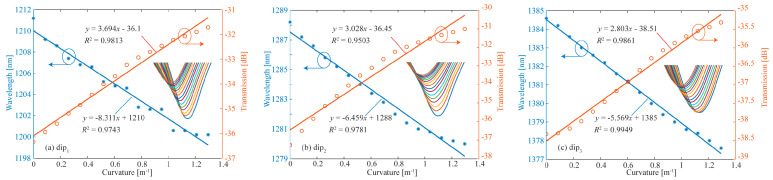
At T = 20 °C, the relationship between curvature and spectral change in dip1 (**a**), dip2 (**b**), and dip3 (**c**) in y-direction.

Similarly, the relationship between curvature and spectral change in dip_1_ (a), dip_2_ (b), and dip_3_ (c) in the y-direction at *T* = 20 °C is investigated. The results are shown in Figure 8. From Figure 8, the curvature sensitivities of three dips are calculated as *k*_1_*_y_* = −3.508 nm/m^−1^, *k*_2_*_y_* = −3.713 nm/m^−1^, and *k*_3_*_y_* = −3.307 nm/m^−1^ in the y-direction, respectively. Compared with the curvature sensitivity in the x-direction, the curvature sensitivity in the y-direction is obviously lower. The reason is that when bending in the x- or y-direction, the inner side of the bent optical fiber is squeezed and the outer side is stretched. Due to the photo-elastic effect, the RI changes on both sides are inconsistent. For the bending in the x-direction, the change in internal and external RI differences caused by bending is greater, so the bending sensitivity is higher.

**Figure 9:**
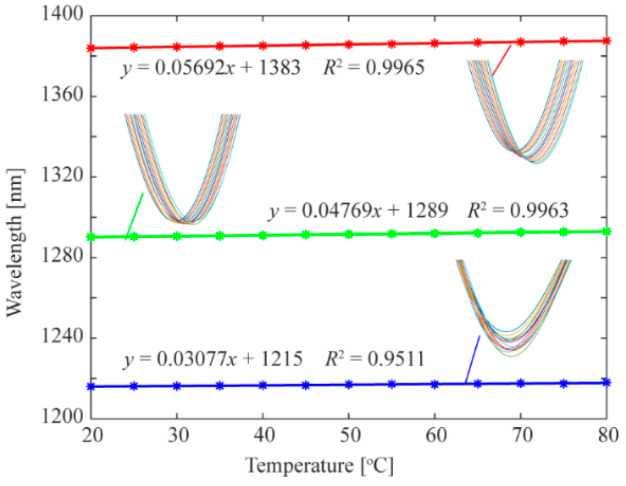
Temperature response curve of dip1 (blue), dip2 (green), dip3 (red). The inset shows their spectral changes.

Afterwards, the influence of temperature change on the sensor also needs to be considered. Under the condition of curvature *C* = 0 m^−1^, the spectral response curve of the sensor at different temperatures (20–80 °C) is shown in Figure 9. With the increase in temperature, the three dips move to longer wavelengths, and the temperature sensitivities are calculated as *k*_1_*_T_* = 0.03077 nm/°C, *k*_2_*_T_* = 0.04769 nm/°C, and *k*_3_*_T_* = 0.05692 nm/°C, respectively. According to the above results, the bending sensitivity and temperature sensitivity components in the coefficient matrix *K* are all obtained. Then, the vector bending measurement with temperature compensation can be calculated by Equations (10) and (11).

**Figure 10:**
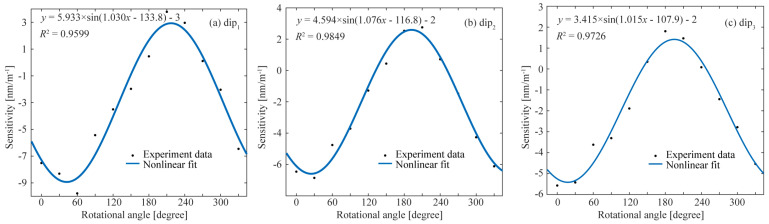
Bending sensitivity of dip1 (**a**), dip2 (**b**), dip3 (**c**) sinusoidal changes with the rotation angle.

In order to further verify the sensitivity differences in the sensors in different directions, the spectral response of vector bending is experimentally studied by changing the bending direction from 0° to 330° in a step of 30°, and the results are shown in Figure 10. From Figure 10, it can be observed that the curvature sensing sensitivity of the three interference dips varying with the bending angle meets the sinusoidal curve. The three data curves have good goodness of fit, and the coefficients of determination are 0.9599, 0.9849, and 0.9726, respectively. The results are in good agreement with Equation (7), and the bending sensitivity changes sinusoidally with the bending angle. For each data curve, their initial phases are not the same. The reason may be that there is an initial angle in the curvature sensing test in different rotation directions. Moreover, there is a direct current bias in all three sinusoidal data curves. The reason is that the SMFs on the left and right sides of the DCF are not in the same plane, but there is an angle.

## 4. Discussion

Furthermore, Table 1 compares various bending sensors exhibiting different sizes, direction discrimination, and curve sensitivities. There are several recent typical bending sensing schemes based on optical fiber. In addition to the characteristics of direction recognition and temperature compensation, the bending sensor proposed in this paper also has high sensitivity and compact size. The performance of bending sensors is comprehensively improved.

The sensor proposed in ref. [12] has higher bending sensitivity, but it has a longer size. On the one hand, it is difficult to use in some environments with limited size. On the other hand, overall, the bending sensitivity per unit length of the sensor proposed in this paper is higher. The sensitivity per unit size of the sensor in this paper is −2.77 nm/(m^−1^·mm), which is higher than that of −0.27 nm/(m^−1^·mm) in ref. [12] (23.085 nm/m^−1^/85 mm). Moreover, it has a temperature compensation function, while ref. [12] does not have temperature compensation and requires an additional temperature sensor in practical applications, which increases the system complexity.

From the perspective of the manufacturing process of sensors, the preparation of the sensor only requires the misalignment fusion splicing of SMF and DCF, without the need for special equipment (such as FBG writing system, femtosecond lasers), and the fusion splicing process takes a short time; the material cost of a single sensor is much lower than that of FBG sensors and multi-core fiber sensors. Therefore, the sensor proposed in this paper has a high cost–performance ratio and is very suitable for occasions with limited costs but high sensitivity requirements.

## 5. Conclusions

A temperature-compensated vector bending sensor with DMZI is proposed and investigated. A modal interferometer composed of a core mode and cladding mode is established by means of the misaligned fusion of a section of DCF and SMF. Owing to the presence of inner cladding of DCF, the sensor experimentally achieved vector bending sensing with temperature compensation. It has different bending test sensitivities in different directions, up to −8.311 nm/m^−1^. Due to its good performance, simple manufacturing process, and low cost, the proposed vector bending sensor has good potential for accurate curvature measurement in the field of bionic robot design.

## Figures and Tables

**Table 1:** Comparison of characteristics between our work and other reported sensors.

Refs.	Cur. Sens. [nm/m^−1^]	Direction	Size [mm]	Temperature Compensation
[12]	−23.085	Sensitive	85	No
[16]	−1.8	Sensitive	20	Yes
[18]	4.362	Insensitive	20	No
[20]	0.01	Insensitive	10	No
Our work	−8.311	Sensitive	3	Yes

## Data Availability

The raw data supporting the conclusions of this article will be made available by the authors on request.
